# Relational thinking and relational reasoning: harnessing the power of patterning

**DOI:** 10.1038/npjscilearn.2016.4

**Published:** 2016-05-11

**Authors:** Patricia A Alexander

**Affiliations:** 1 Department of Human Development and Quantitative Methodology, University of Maryland, College Park, MD, USA

## Abstract

This article offers an overview of the nature and role of relational thinking and relational reasoning in human learning and performance, both of which pertain to the discernment of meaningful patterns within any informational stream. Distinctions between thinking and reasoning relationally are summarized, along with specific forms of patterning that might be discerned. Next, the article summarizes what is presently known about relational reasoning, and then moves to explore future directions in educational research and in instructional practice that warrant attention based on the empirical literature.

## Introduction

A baby reaches for his mother among a gathering of women; a student becomes enrapt in a story, seeing herself in the main character; an attending physician realizes that her patient is displaying abnormal symptoms indicative of acute myocardial infarction; and a physicist sets out to disprove an espoused cosmological theory. At the core of all human learning and performance, as with the diverse episodes just described, is the foundational ability to perceive patterns that thread through all of nature, including human nature. Those patterns can be as intimate as a hand gesture;^1^ as academically core as literacy comprehension;^2^ as critical as effective medical diagnosis;^3^ or as sweeping as the laws of the physical universe.^4^

Without the ability to discern meaningful patterns in the stream of data that continually flood the senses, humans would remain prisoners within a world of isolated sights, smells, and sounds, unable to comprehend or to build on experiences across time and space. Thankfully, humans enter the world with the capacity to perceive patterns within the sensory information that surrounds them and then draw on that capacity in intentional, effort, and strategic ways to promote higher-order cognitive processing.^5–7^ Granted that initial capacity, which we labeled as relational thinking,^8^ can be quite primitive and can vary greatly from person to person or from situation to situation, but it is nonetheless the neurobiological functioning that guides the development of human perception and cognition across the lifespan.

This contention that the ability to discern patterns within any informational stream is rudimentary, pervasive, and essential is by no means new. From the philosophical writings of Heraclitus, Aristotle, and Immanuel Kant to William James and John Dewey, from the Gestalt school of psychology to contemporary research in cognitive science^9,10^ and neuroscience,^11,12^ the foundational nature and potency of relational thinking appears undeniable. Yet, there is still much to be learned about pattern perception and its purposeful utilization. Toward that end, what new insights are offered herein pertain to the emerging body of psychological, cognitive neuroscience, and psychometric research on the character of the relations that might be spontaneously perceived (i.e., relational thinking) and, more particularly, on the intentional harnessing of pattern recognition to drive higher levels of human learning and performance (i.e., relational reasoning).

## Thinking and reasoning relationally

To move forward in the discussion of harnessing the power of patterning, it is important to first disentangle the two associated processes I have referenced, relational thinking, and relational reasoning—processes that operate in concert to allow for the coupling of percepts and concepts. Further, it is essential to consider how those notions compare with associated concepts that populate the cognitive science and neuroscience literatures.^13–15^

### Relational thinking and reasoning in comparison

According to Peirce,^16^ percepts, which are mental impressions formed in the moment from the sensory systems data,^17–19^ are “the starting point of all our reasoning” (p 308). Percepts are not isolated, occasional, or singular occurrences. Rather, at any given moment, minds are being bombarded by innumerable percepts.^20,21^ Further, those percepts continue unabated, regardless of human will, judgment, or knowledge.

In fact, these configurations are, for the most part, fleeting sensations, remaining largely outside of human awareness,^20^ that is, unless those percepts garner attention or become consciously accessible.^22^ It is through relational thinking that the onslaught of perceptions becomes recognizable or consciously accessible as some discernible object or idea (i.e., concepts). Without relational thinking, the innumerable percepts would remain separate pieces and never assemble into impressions or rudimentary forms that could potentially influence human thought or action. In effect, without relational thinking, there is no mechanism for building on percepts or for the coupling of those percepts with the concepts that populate the human mind.^8^

Nonetheless, more is required of human performance than a reliance on more instinctual, spontaneous, and fleeting discernments of patterns (i.e., relational thinking). For the attainment of more sustained, deeper, and what are popularly regarded as higher-order forms of cognitive thought and performance, humans must build on the more innate capacity to perceive patterns. The mechanism that my colleagues and I have targeted that serves the fundamental need to purposefully harness the power of patterning is relational reasoning.^8,23^

Although it is important to acknowledge that the boundaries between these more intuitive and intentional systems of mental processing may not be categorically distinct,^24^ I nonetheless juxtapose these two forms of pattern recognition on several key dimensions, including locus, temporal frame, and cognitive demands to sharpen the salient contrasts (Table 1). As the comparison offered in Table 1 suggests, relational thinking can, thus, be characterized as more fleeting, external, and rather effortless and unconscious in nature. This stands in contrast to relational reasoning, which has a more enduring, representational quality and which demands effort and intentionality on the part of the human mind. Although a more in-depth consideration of the neurobiological underpinnings of relational reasoning is beyond the scope of this overview, there is ample evidence that this capacity, which emerges within the first years of life, rapidly develops into middle and late adolescence.^11,25^ Further, particular regions of the brain, most notably the rostrolateral prefrontal cortex, seem especially implicated when children and adult engage in relational reasoning tasks.

### Relational thinking and reasoning in perspective

With the brief comparison of relational thinking and relational reasoning as a backdrop, let me situate those characterizations within the broader literatures in cognitive science and neuroscience that touch upon such underlying mental capacities and processes. For one, the distinction my colleagues and I have drawn between the more intuitive versus the more intentional systems of relational processing is not commonly addressed within the cognitive science and neuroscience literatures. Rather, a more consolidated focus on higher forms of cognition predominates, and understandably so given the emphasis on relational processes within sophisticated problem-solving areas such as mathematics, rational thought, and scientific reasoning.^13,26^ For example, in their review of relational knowledge, Halford *et al.*^27^ (p 488) focused on “relational representations”, which they distinguish from more automatic, modular, or nonanalytic processes.

When the discussion progresses to more intentional and effortful relational processes, the similarities and differences that arise between the conceptualizations and operationalizations offered herein and those populating the cognitive science and neuroscience literatures are more intricate and nuanced. At the conceptual level, for instance, the definition of relational reasoning framing my colleagues’ and my program of research is generally compatible with that associated with the neuroscience literature. For instance, Krawczyk *et al.*^28^ (p 588) characterize relational reasoning as the human brain’s “unique capacity to reason about abstract relationships among items in our environment”—a conception that parallels our characterization of relational reasoning as the ability to discern meaningful patterns in the stream of data.^23^ To this base definition, others^11,27^ make explicit reference to relations between “mental” representations. Although multiple representations are involved in our conceptualization and operationalization of relational reasoning as well, those representations may be *in mundi* as well as *in mente*—a subtle but relevant distinction that allows for the percept–concept coupling with which I previously eluded.

Another similarity between the conception of relational reasoning evoked in our current program of research and that populating the cognitive science and neuroscience literatures centers on executive function factors such as working memory and inhibitory control that seemingly underlie this and other forms higher cognition.^29–31^ Further, depending on the nature of the symbols entailed in *in mundi* or *in mente* representations (e.g., linguistic, numeric, figural, or graphic), additional individual differences factors such as visuospatial memory, reading fluency, and domain-specific knowledge can prove influential to the discernment of meaningful patterns within informational displays.^27,32–34^ Moreover, when relational reasoning is examined within novel problem-solving tasks or contexts, it is indicative of fluid intelligence.^25,35,36^

As the noted similarities suggest, much of the essence of relational processing represented in the cognitive science and neuroscience literatures is preserved in the theoretical and empirical work my colleagues and I have undertaken. However, what my colleagues and I have sought to contribute to the discourse pertains more directly to the manifestations of relational reasoning being explicitly explored by cognitive scientists and neuroscientists, and the manner in which those manifestations are systematically investigated. Specifically, as will be elaborated in the ensuing sections, my colleagues and I have attempted to push the exploration of relational reasoning beyond its more routine foci so as to consider multiple forms, measures, and techniques that can be utilized to unearth the varied forms of this foundational mental capacity.

Toward that end, I will now turn to the specific forms and processes associated with relational reasoning that have been the focus of theoretical and empirical work by my colleagues and me, as well as by others (e.g., references 37–39). Subsequently, I will attempt to outline what my colleagues and I have come to learn about the relational reasoning, and what remains to be understood. Although not seeking to diminish the efforts underway in the broader research communities, I will highlight the recent work my collaborators and I undertaken in this brief survey. I will also consider how relational reasoning manifests in everyday functioning and problem solving, and what steps can be taken to harness that nature in service of fostering learning and academic development.

## Relational reasoning in form

Over the past 4 years, my colleagues and I have delved into the construct of relational reasoning for the purpose of finding ways to ascertain its nature and to gauge its role in human learning and performance. Among our initial realizations was that much of the recent work in cognitive science and neuroscience, while theoretically informative, did not entirely serve the needs of educational researchers for several reasons.^29^ For one, the methods employed to examine relational reasoning within cognitive science and neuroscience are highly specialized (e.g., functional magnetic resonance imaging or event-related potential) and cannot be pragmatically or widely utilized in educational research. Second, although the term relational reasoning as applied within these fields is similarly defined in terms of pattern perception, only one form of such pattern perception is routinely examined (i.e., analogical reasoning^35^).

Further, the research in neuroscience demonstrates an overreliance on a singular measure, the Raven’s Matrices,^40^ thereby restricting examination to only one form (analogical reasoning) and only one mode of representation (figural^29,35^). Thus, in our research, we sought to investigate multiple forms of relational reasoning and to devise multiple psychometrically sound measures of relational reasoning entailing both figural and linguistic representations. We also wanted measures that could be easily administered to children, adolescence, and adults either online or in print. Samples of items from two of the resulting measures suitable for older adolescents and adults—the Test of Relational Reasoning (TORR^41–43^), and the Verbal Test of Relational Reasoning (vTORR^44,45^)—are provided in the Supplementary Appendix.

As the sample items in the Supplementary Appendix illustrate, the TORR is figural in form and intended to function as a more fluid measure of relational reasoning ability. In contrast, vTORR was conceived as a somewhat more crystallized test of relational reasoning due to its linguistic content, although the novelty of the items still entail fluid or flexible problem solving on the part of respondents. A third measure, the Test of Relational Reasoning-Junior (TORRjr^46,47^) was developed for use with children and early adolescents. The TORRjr was devised to be an easier version of the TORR and as such parallels that measure in its scales, items, and overall format.

Drawing on the extant literatures in reasoning (e.g., reference 48), mathematical set theory (e.g., reference 49), and philosophy (e.g., reference 50), my colleagues and I ultimately settled on four forms of relational reasoning that we felt encompassed key patterns of similarity and dissimilarity that could be discerned within any informational stream.^23,35^ Those four forms, as illustrated in the sample items in the Supplementary Appendix, pertained to patterns of similarity (analogical reasoning), discrepancy (anomalous reasoning), opposition (antithetical reasoning), and exclusivity (antinomous reasoning). Although other forms of relational reasoning likely exist, these four forms were found to have theoretical and empirical grounding within the educational, psychological, and philosophical literatures.

Such grounding is especially apparent for the empirical investigations of analogical reasoning, which has garnered the most attention in the explicit study of relational reasoning (e.g., references 10,51), and anomalous reasoning, which has focused largely on the domains of science and mathematics (e.g., references 52,53). For example, Hofstadter^54^ (p 499) has described analogies as “the very blue that fills the whole sky of cognition”. With similar conviction, Chinn and Brewer^38^ (p 1) contended that “understanding how science students respond to anomalous data is essential to understanding knowledge acquisition in science classrooms”, as well as how students undergo theory change more broadly.

Although the empirical evidence for antinomous and antithetical reasoning may be somewhat less prevalent, it exists nonetheless. For instance, the research dealing with ontological categories within the sciences, especially the biological science (e.g., alive versus not alive; animal or plant), requires individuals to reason antinomously.^37,55^ These very notions of antinomous and antithetical reasoning can be found within the writings of the pre-Socratic philosopher, Heraclitus, who wrote about the unity of opposites.^56^ In essence, what Heraclitus contended was that we can only really come to know something through its relation to its true opposite. At first glance, Heraclitus would seem to be making a case for antithetical reasoning. However, his discussion of “true” opposites takes on a more paradoxical orientation. In effect, to know happiness, we must juxtapose it to “not happiness”, or to understand “good” there must be “not good”. Support for more antithetical orientations can be found in the substantial literatures dealing with persuasive text and with conceptual change that consistently indicate the power of reasoning about opposing views or counterarguments to improve comprehension^57,58^ and to dismantle misconceptions within academic domains.^59–61^

### *In vitro* studies

Over the past 5 years, my colleagues and I have sought the empirical evidence of these four forms and examined the association between relational reasoning and performance in varied cognitive domains. These empirical investigations have been both *in vitro* and *in vivo* in nature. For the *in vitro* studies (i.e., laboratory or experimental research), we submitted the previously described measures of relational reasoning (i.e., TORR, vTORR, and TORRjr) to various analyses within a number of academic domains. These analyses were undertaken to establish the psychometric properties of these measures, examine item functioning, determine underlying factor structures, and test differing structural models of relational reasoning. We also explored the association between relational reasoning and select executive function and individual difference indicators (e.g., comprehension ability and visuospatial working memory).

What this collection of investigations has revealed is that these three formal measures are psychometrically sound assessments of relational reasoning with items that operate within acceptable difficulty parameters (i.e., references 30–70) and that factor as expected. Further, we have ascertained that visuospatial working memory and reading comprehension ability were only moderately associated with the performance on TORR and vTORR, respectively.^42,43,45^ We also tested the degree of association between TORR and the Raven’s Matrices,^40^ which is so commonly used in neurobiological studies of relational reasoning. We determined that there was a significant positive correlation between these two presumed measures of relational reasoning, and that the TORR was more difficult for participants than the Raven’s.^42^

One recent investigation by Grossnickle *et al.*^62^ used items from the TORR to explore the componential processes underlying the four forms of relational reasoning via Bayesian network analyses. Grossnickle *et al.* found that the component processes of encoding, inferring, mapping, and applying that Sternberg^48^ ascribed to analogical reasoning were also evident in students’ processes of anomalies, antitheses, and antinomies. These researchers also determined that low-performing students struggled more with working memory demands at the point of inferring and mapping.

There has also been evidence of significant associations between TORR scores and performance in the domains of engineering design^63^ and maternity nursing (Fountain^64^). For example, Dumas and Schmidt^63^ determined that those with higher TORR scores, especially for the antinomy scale, produced more creative solutions to engineering design problems. Similarly, Fountain^64^ found that relational reasoning capacity, as measured by the TORR, was a significant predictor of maternity nurses critical thinking as measured by their analysis of medical cases.

### *In vivo* studies

Alongside these more experimental investigations, we have been exploring relational reasoning *in vivo*, that is, within naturally occurring settings that involve complex problem solving. These studies have uncovered evidence of analogical, anomalous, antithetical, and antinomous reasoning in the interactions between an attending physician and resident physicians engaged in diagnosing and treating patients.^3^ In this investigation, Dumas *et al.* also demonstrated how these various forms of relational reasoning worked in concert to lead to more effective clinical outcomes. Jablansky *et al.*^65^ similarly identified occasions of relational reasoning as first through twelfth graders thought aloud about the form and function of more or less familiar technological tools. What was significant about this study was not only the manifestation of all forms of relational reasoning even among the youngest students but also the differences in the quantity and quality of reasoning associated with the grade level and object familiarity.

## Relational reasoning in principle

Together, the *in vitro* and *in vivo* investigations of relational reasoning just overviewed have contributed to certain insights about its nature and its importance to human learning and development. Recently, my colleagues and I^66^ were asked to share what we have come to learn about relational reasoning with a particular eye toward educational policies. Here I revisit those insights and then subsequently turn to the implications of this emerging literature to next steps in empirical research and instructional practices for all those broadly concerned with human learning and development. I also take the liberty to outline some of the lingering questions that each of these principles about relational reasoning brings to the surface.

Specifically, according to Alexander *et al.*,^66^ the following claims about relational reasoning can be forwarded:

- The ability to reason relationally is foundational and pervasive.
- Relational reasoning can be observed and measured in diverse ways.
- Relational reasoning varies by age, domain, and context.
- Relational reasoning is malleable and teachable.

### Foundational and pervasive

As I have sought to establish from the outset, relational reasoning, especially when coupled with its more intuitive, spontaneous counterpart, relational thinking, underlies all human performance—an observation shared by cognitive scientists and neuroscientists.^10,27,39^ Early in the twentieth century, Spearman,^67^ one of the progenitors of modern intelligence testing and someone strongly influenced by the Gestalt school, came to see human intellectual capacity largely in terms of pattern perception. His search for the unitary intelligence factor “*g*” was orchestrated around certain “fundamental laws”, including the law of the eduction of relations, which pertains to the power to bring relations to mind.

Although I am not seeking to make a case for any “*g*” factor of intelligence, I do see certain parallels between Spearman’s arguments for the essentialness of perception and attention to patterns and the contemporary work on relational reasoning. Simply stated, if individuals cannot perceive and do not attend to the relations embedded within sensory information that continually floods them, then they will undoubtedly be relegated to a world that consists solely of noise or fragmentary pieces of sensory data that carry little or no meaning. For these reasons, relational reasoning is unquestionably a fundamental and pervasive capacity.

Further, this underlying capacity to perceive patterns is sufficiently fluid or flexible to allow for iterations when problems are nested within specific domains (e.g., engineering, mathematics, medicine, or reading). For that reason, and as seen in the studies in the medical professions^3,64^ and engineering,^63^ certain forms of reasoning may be more evident when domain-specific problems are engaged. What cannot be ascertained at this point, however, is the precise nature of interplay between domain-general and domain-specific iterations of relational reasoning forms.

### Observable and measurable

As an empirical researcher, more is required than simply believing in the foundational nature of relational reasoning. What is necessary is to observe and measure relational reasoning in psychometrically sound ways. Through observation, researchers are able to bear witness to relational reasoning’s presence within naturally occurring occasions of reasoning and problem solving, as my colleagues and I have done in eavesdropping on the interactions within a medical team^3^ or children whose technological literacy is being gauged.^65^

By comparison, through measurement, researchers have ascertained the role that relational reasoning has in human learning and performance for a range of situations and contexts. For my colleagues and I, the TORR, vTORR, and the TORRjr have become portals onto the processes and power of relational reasoning. The connections documented between relational reasoning and creative engineering designs^63^ and effective critical thinking in maternity nursing^64^ are two such cases in point. Through the process of establishing the psychometric qualities of these measures, we also found that relational reasoning, although correlated at a low or moderate level with measures of comprehension, visuospatial working memory, and even the Raven’s Matrices, makes significant and unique contributions to cognitive outcomes over and above such well-established indicators.

What is less understood about these processes has more to do with the way in which relational thinking and relational reasoning work together within more dynamic and collective problem-solving situations. We see hints of this interactivity in the *in vivo* studies that shed some light on the process by which the patterning of one individual sparks the relational processing of others.^3^ For instance, when a medical resident notes a particular anomaly in the symptoms of a patient, there is a greater likelihood that other residents will interject additional anomalies into the discussion. Or, when the attending physician, a recognized expert, reminds the residents of an analogous case, the direction of the diagnosis shifts and new similarities and differences are introduced into the discourse. Of course, much more needs to be explored about dynamic and collective problem-solving contexts and the influence that these contexts exert on the flow of relational reasoning.

### Age, domain, and context dependent

Employing both observations and measures, we have come to learn that the fundamental and pervasive character of relational reasoning does not translate into uniformity across ages, domains, or contexts. Nowhere is this more evident than in the cross-sectional data that Jablansky *et al.*^65^ gathered for students in first to twelfth grade. For one, these researchers found that although relational reasoning occurred at all these grade levels, younger students required more external support or scaffolding than older students. In addition, among younger students, there were more occasions of analogical and anomalous reasoning and relative fewer instances of antithetical and antinomous reasoning. Conversely, the older students relied more on antithetical and antinomous reasoning rather than on analogical and anomalous reasoning. Finally, Jablansky *et al.* determined that the problem-solving context mattered. Specifically, there were significantly more occurrences of analogical reasoning over the other four forms when the objects being analyzed for their form and function were familiar rather than unfamiliar.

Why these developmental shifts in reasoning patterns would arise is a question worthy of further exploration. At this point, there is reason to speculate that some of the shifts occurred because of the children’s increased knowledge and experiences—in this instance about various technologies and their functions. However, increased knowledge alone does not account for the greater reliance on antitheses and antinomies among the older students in this investigation. Perhaps some of the explanation lies in the neurobiological changes that occur in middle and late adolescence—changes that are seen to support relational reasoning capability.^11,25^

### Malleable and teachable

Just as intelligence has been shown to be changeable as a consequence of relevant experiences,^68,69^ relational reasoning should likewise be regarded as malleable and teachable. However, the degree of malleability or teachability remains open to debate. For instance, as evident in the cross-sectional study of students from grades 1 to 12 by Jablansky *et al.*,^65^ there were apparent quantitative and qualitative shifts in relational reasoning that occurred from childhood and into adolescence, even in the absence of any explicit training of these reasoning forms. By contrast, Fountain^64^ found no significant change in TORR performance for maternity nurses at prelicensure through to those with >10 years of experience. Such an outcome suggests relatively stability in the level of relational reasoning after late adolescences when no cognitive maturation, pertinent experiences, or direct interventions were implicated.

Moreover, there is ample evidence that analogical reasoning performance can be directly trained, including the studies my colleagues and I have conducted with children and adults.^70–72^ Others have documented similar effects for interventions involving analogical reasoning.^39^ The question remains whether similar training outcomes to those documented for analogies would be expected for the other forms of relational reasoning, anomaly, antithesis, and antinomy.

In effect, would we anticipate that individuals’ overall relational reasoning performance could be either directly or more indirectly manipulated? On the basis of certain evidence, my response to that question is “yes”. For one, there is now empirical evidence that the component processes of analogical reasoning (i.e., encode, infer, map, and apply^48^) that my colleagues and I employed in the training of this form of relational reasoning^72^ also underlie anomalous, antithetical, and antinomous reasoning.^62^ Thus, it is reasonable to assume that these same component processes can be used as the basis for training relational reasoning more broadly.

Further, it has been shown that relevant interventions that do not expressly target the four forms can produce shifts in TORR scores. Such indirect effects were apparent in Dumas and Schmidt’s^63^ study of engineering design students. These researchers found that students’ exposure to a design intervention resulted in an even stronger association between creative engineering design solutions and relational reasoning, especially for antinomous reasoning. Others are presently exploring the effects that interventions aimed at critical analytic thinking on both written arguments and oral discussions.^73,74^ For instance, students in the Murphy *et al.*^74^ study were trained to formulate elaborated explanations in writing, which are detailed justifications for claims made. Although all four forms were identified in students’ written products, instances of analogies and antitheses were more prevalent. Similarly, when training high-school physics and chemistry students to engage in exploratory talk (i.e., two or more people exchanging responses around a provocative question or issue), Greene *et al.*^73^ documented frequent occasions of antithetical and anomalous reasoning. Both of these investigations illustrate fruitful approaches for enhancing students’ relational reasoning when focused primarily on improving the quality of written or oral argumentation skills.

Although I am fairly confident in the teachability of relational reasoning to some level, there are lingering questions as to the form that any explicit training should take, especially with regard to the domain specificity of the intervention. What the rich literatures on strategy or problem-solving training suggest, however, is that efforts that are entirely generic in form are less likely to have lasting effects.^75^ Thus, embedding such training within a knowledge domain and aligning it with problems and tasks that are central to that domain—be it medical diagnosis, mathematical problem solving, or reading comprehension—seems more likely to promote immediate and enduring effects.

## Future directions for research and practice

Given what the field has come to understand about the nature and importance of relational reasoning, the question remains as to what lies ahead for this field of inquiry. Let me identify several directions that appear especially promising in light of known and emerging findings about relational reasoning.

### For research

There are several obvious avenues for research in relational reasoning that need to be actively pursued to elaborate on and extend what I have outlined in this overview. Those avenues pertain to: (a) longitudinal examinations; (b) studies that incorporate physiological or neurological data; and (c) cross-cultural and cross-context investigations. For example, to date the construction of relational reasoning’s developmental trajectory has relied largely on cross-sectional research. Thus, it is imperative to undertake more longitudinal investigations of relational reasoning, especially ones that encompass points of significant cognitive, neurobiological, experiential, and psychosocial transition such as that marked by the movement from middle school into high school or into college. Such longitudinal studies could be incorporated into ongoing studies of expertise development as well, in order to ascertain whether transitions from acclimation into competence or expertise are accompanied by concomitant transformations in relational reasoning capacity or performance patterns.

As I noted, much of the work on relational reasoning within neuroscience has looked exclusively at analogical reasoning^3,29^ and has relied extensively on items from the Raven’s Matrices.^40^ Consequently, what is not understood is whether the engagement in anomalous, antithetical, or antinomous reasoning would produce similar brain activation patterns to those documented for analogical reasoning. As all four forms apparently share underlying componential processes, and are moderately correlated,^62^ there is reason to hypothesize that they operate in neurologically similar ways. Yet, there is also cause to presume that there is sufficiently neurological variability, especially for the processing of antinomies, which demand the establishment of exclusion between two sets of information.

Finally, the tests that my colleagues and I have devised were intended to serve as more novel or fluid measures of relational reasoning capacity.^42^ That is even true of the vTORR, which relies on linguistic information but still entails the performance of non-traditional reasoning tasks. At present, we do not know whether there is measure non-invariance for any of these tests for different ethnic or gender groups, although the ongoing study by Dumas^76^ on the TORR has found no evidence of non-invariance at the item level. Similarly, the international studies currently underway in Israel and New Zealand are expected to shed light on any cultural differences that might manifest on the TORRjr. But much more needs to be learned about how relational reasoning might iterate not only within diverse cultures but also across varied contexts such as in professional practices like medicine, nursing, or engineering and the complex problem solving they involve.

### For practice

Within the educational and psychological research communities to which I belong, there are certainly avenues that merit exploration related to practice. Those avenues include: (a) predictive studies that explore the use of relational reasoning as a measure to identify academic potential; (b) classroom-based investigations that seek to expressly train relational reasoning within such a dynamic environment; and (c) domain-specific studies that explore the enactment of relational reasoning for contrasting fields such as physics or history. I raise the notion of predictive studies because one of my primary purposes for embarking on the study of relational reasoning and the development of relevant measures was to forge tools that could lead effectively to the identification of fundamental cognitive capabilities that might otherwise be overlooked by more traditional screening measures. Will performance on the TORR signal unrecognized potential missed by traditional crystallized measures of achievement or aptitude? In what way will performance on the TORR or one of its iterations unearth future success in academics or in later professional practice? To what extent do students with identified learning or cognitive problems perform differently on the TORR, vTORR, or TORRjr compared with their non-identified peers? Ultimately, having psychometrically sound measures of relational reasoning is only the first step toward addressing such important questions.

This issue of screening for academic potential raises another ethical concern regarding relational reasoning. To be more precise, if relational reasoning is a foundational and pervasive capability and if relational reasoning can be trained or improved, then is there no obligation to train individuals to reason better analogically, antithetically, antithetically, and antinomously? This has long been my personal position. For that very reason, my colleagues and I are committed to articulating models for relational reasoning training based on the componential processes we have utilized in the past.

As mentioned, one of the lessons that I learned from those prior forays into classroom-based training is that the processes of reasoning relationally cannot be treated generically, that is, relational reasoning training should not be isolated from the content with which students are typically or routinely engaged. Rather, it makes sense that relational reasoning be naturally nested within the academic domains that frame the educational experience for students. Thus, future efforts to enact relational reasoning within learning environments demand that educators recognize the place of analogies, anomalies, antitheses, and antinomies in the subjects they teach—whether those subjects are reading, history, mathematics, or science.

Further, teachers must themselves be familiar and comfortable with all forms of relational reasoning and their manifestations in the content of schooling. Likewise, educators at all levels of educational practice must become models of relational reasoning. Those who do not reason relationally, as a habit of mind, or who do not engage in relational reasoning, as a course of action, cannot be expected to promote relational reasoning in those whose academic development they seek to foster. Consequently, it would seem that the path to improved relational reasoning in students must pass through the teachers and the educational systems that those teachers and their students inhabit.

## Conclusions

It was my goal in this treatise to introduce the reader to the construct of relational reasoning and to grapple with the way in which relational reasoning may be manifested and measured. The past 5 years have been replete with discoveries and insights about this foundational capacity to find meaningful patterns within the deluge of information that washes over us all. Although there is unquestionably more to be discovered about relational reasoning, I feel that the field has garnered sufficient evidence to move to action. In effect, it is the time to put the knowledge of relational reasoning to work, to harness its potential in service of improved learning and development.

## Figures and Tables

**Table 1:** Comparison of relational thinking and relational reasoning

*Attribute*	*Relational thinking*	*Relational reasoning*
Locus	*In mundi* (world)	*In mente* (mind)
Temporal frame	In the moment	Over time
General nature	Largely impressionistic; felt or sensed	Capable of being examined or justified
Cognitive demands	Effortless, unconscious	Effortful, consciously evoked
Relational forms	Predicated on discernment of similarities and dissimilarities	Predicated on discernment of similarities and dissimilarities
