# Supplementary material for: Relational thinking and relational reasoning: harnessing the power of patterning
Source: NPJ Sci Learn. 2016 May 11;1:16004. doi: 10.1038/npjscilearn.2016.4 (PMC6380381; doi:10.1038/npjscilearn.2016.4)
Supplement: Supplementary Appendix [file npjscilearn20164-s1.doc]

**APPENDIX**

**Relational Reasoning Figural (TORR) and Verbal (vTORR) Sample Items**

**Analogy**


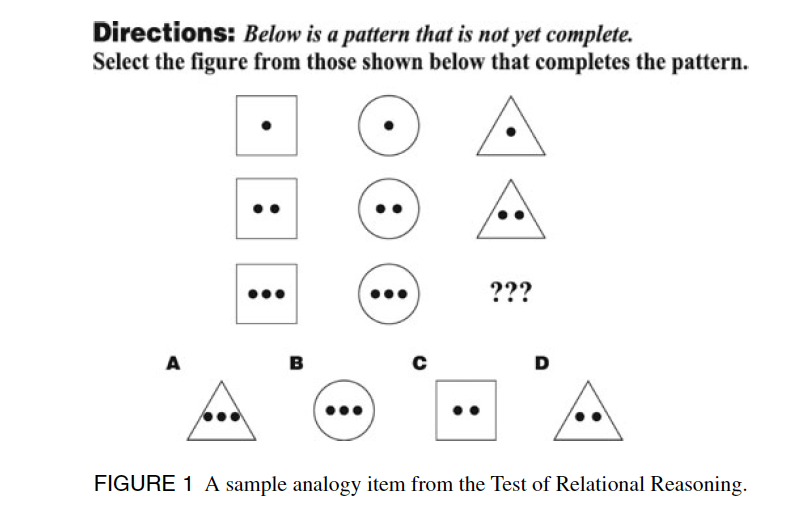


**Directions**: Select the sentence from the answer choices below that describes the ***most similar*** situation.

The man breathed a sigh of disappointment when he opened his wife’s gift.

1. The boy felt saddened as he packed up his old clothes to hand down to his younger brother.
2. The mother couldn’t hide her sense of defeat when she received her child’s report card.*
3. The girl felt an immediate sense of relief after getting an invitation to the dance.
4. The child’s bafflement showed when his friend didn’t share his dessert with him.

*the correct response.

**Anomaly**

**
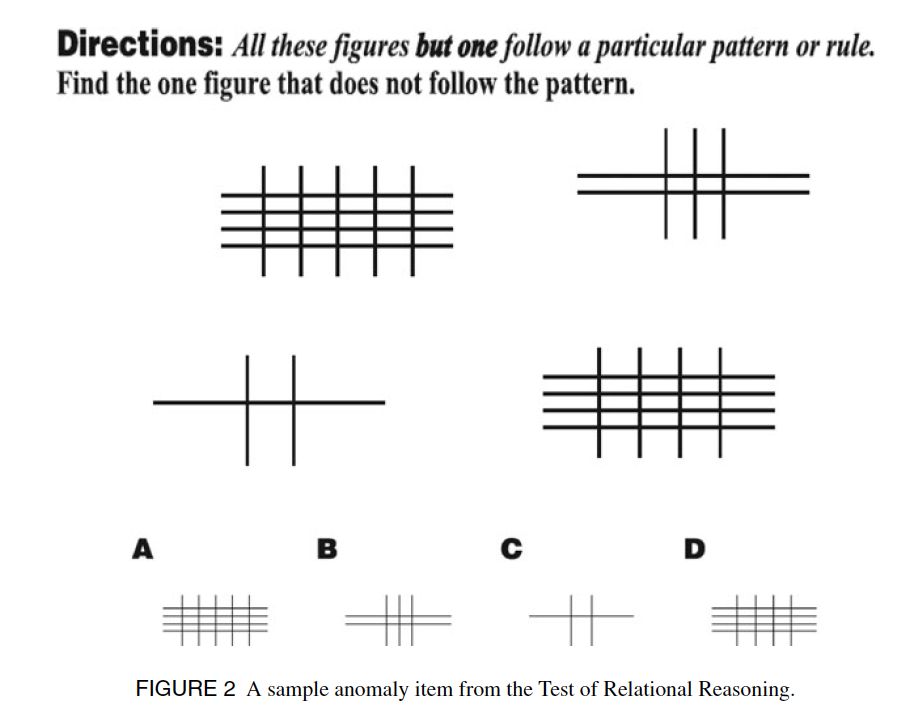
**

**Directions:** Three of these sentences follow a particular pattern or rule. Find this pattern or rule and select the sentence that does not follow the pattern.

1. The pride of lions devoured a wildebeest.
2. The school of piranhas feasted on the bird.
3. The herd of buffalo munched on the grass.*
4. The pod of killer whales gobbled up a seal.

*the correct response.

**Antimony**


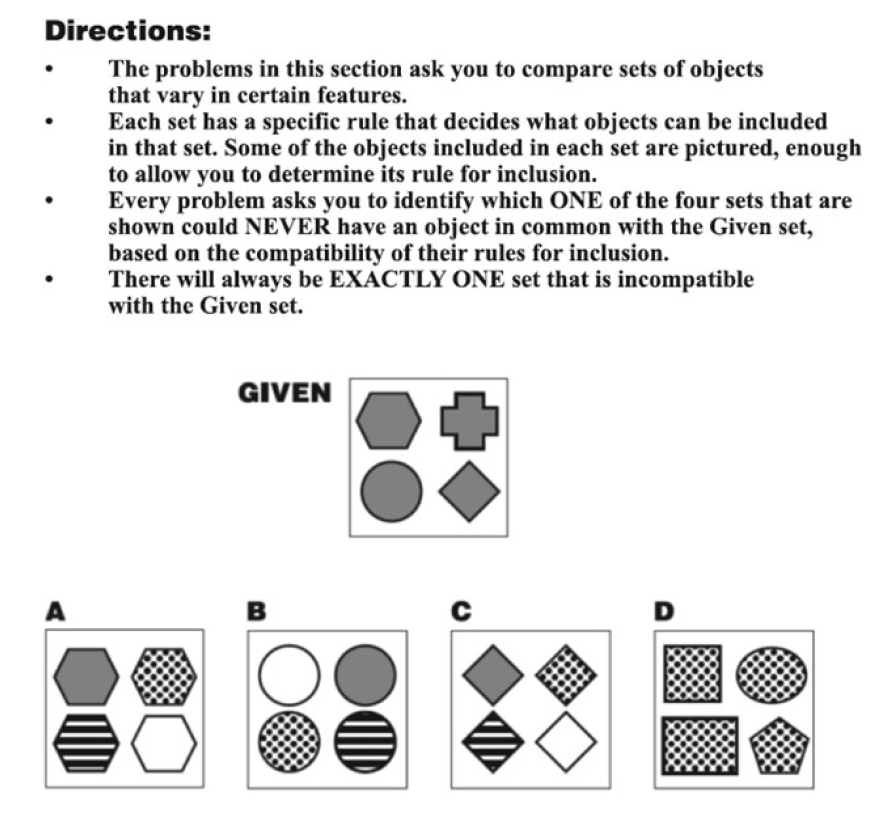


**Directions**: Read the two paragraphs below. Then select the sentence that includes ***an idea that could be reflected in one paragraph but not*** ***the other***.

Running is a great way to get in shape and relieve stress. Long runs leave me feeling energized and excited for the day. They also boost my self-confidence and make me feel like I can accomplish anything.

Team sports provide opportunities to spend time with friends, with the added benefit of exercise! I enjoy bonding with my friends during a Saturday morning soccer game or Wednesday night kickball tournament.

A. Exercise is valuable for your body.

B. Running can be exhilarating.

C. Exercise should be done socially.*

D. Kickball isn’t really a sport.

*the correct response.

**Antithesis**


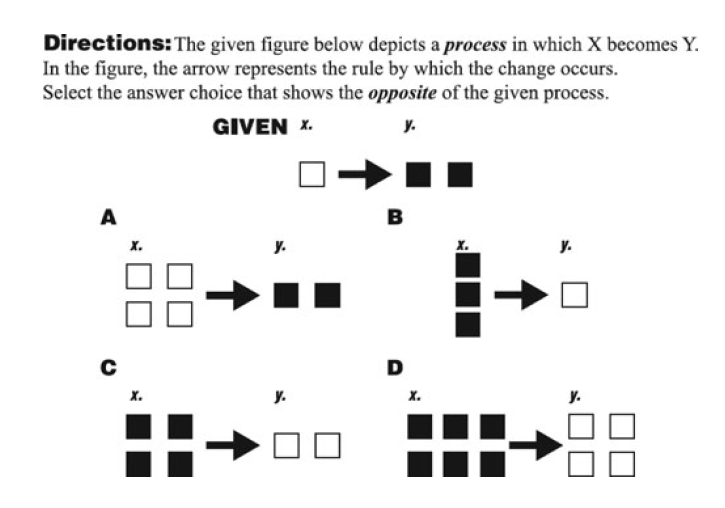


**Directions:** The sentence below describes a situation. Select the sentence from the answer choices below that describes the ***opposite*** situation.

The woman was pleased with the dollhouse she built.

A. The child was satisfied with the blocks he had stacked into a tower.

B. The boy was amused when he broke his dinner plate.

C. The girl was frustrated that she had to put together a bookshelf.

D. The man was angry that he destroyed his painting.*

*the correct response.
